# Prosthetic Aortic Valves: Challenges and Solutions

**DOI:** 10.3389/fcvm.2018.00046

**Published:** 2018-05-14

**Authors:** Lucia Musumeci, Nicolas Jacques, Alexandre Hego, Alain Nchimi, Patrizio Lancellotti, Cécile Oury

**Affiliations:** ^1^Laboratory of Thrombosis and Hemostasis and Valvular Heart Disease, GIGA Cardiovascular Sciences, University of Liège Hospital, CHU Sart Tilman, Liège, Belgium; ^2^Department of Cardiology, GIGA Cardiovascular Sciences, University of Liège Hospital, Heart Valve Clinic, CHU Sart Tilman, Liège, Belgium; ^3^Gruppo Villa Maria Care and Research, Anthea Hospital, Bari, Italy

**Keywords:** aortic valve replacement, mechanical valve, bioprosthesis, percutaneous, surgical, complications

## Abstract

Aortic Valve Disease (AVD) is the most common Valvular Heart Disease (VHD), affecting millions of people worldwide. Severe AVD is treated in most cases with prosthetic aortic valve replacement, which involves the substitution of the native aortic valve with a prosthetic one. In this review we will discuss the different types of prosthetic aortic valves available for implantation and the challenges faced by patients, medical doctors, researchers and manufacturers, as well as the approaches that are taken to overcome them.

## Introduction

In Europe alone more than 13 million people (1) are diagnosed with Valvular Heart disease (VHD) each year and 100 million worldwide (2). VHD primarily affects the elderly (>65 years old) in western countries and young people (<30 years old) in developing countries, because of the high incidence of rheumatic heart disease and short life expectancy (3 ESC Guidelines; Supplement).

The deterioration of native heart valves (tricuspid, pulmonary, mitral, aortic) once started is difficult to treat or revert with medications, leaving valve replacement as the only option, whenever valvuloplasty is not possible (4).

Aortic Valve Disease (AVD) is the most common among valvular conditions (44,3% VHD are AVD) (5) and the gold standard treatment was Surgical Aortic Valve Replacement/Implantation (SAVR or SAVI) until the introduction in 2007 of a new revolutionary procedure, Transcatheter Aortic Valve Replacement/Implantation (TAVR or TAVI). TAVI became especially used in inoperable, *i.e.*, high-risk patients, as it is less invasive than an open-heart surgery (6).

Both SAVI and TAVI are not risk-free, tough, in fact, patients are subjected to life threatening complications associated with the medications given post-implantation and with the deterioration of the implanted valve (7).

In this review we will discuss prosthetic aortic valves, pre and post implantation challenges, and their solutions.

### Aortic Valve Disease (AVD)

Aortic Stenosis (AS) accounts for the majority of AVD (almost 50% of all VHD). AS prevalence in Europe is 3–8% among people over 75 years old. If untreated, 90% of patients with severe AS have a life expectancy of less than 10 years, and 50% of the patients will die in the 2–3 years following symptoms onset (3 ESC Guidelines; Supplement).

Calcific Aortic Stenosis (CAS), which is the formation of fibro-calcic nodules on the valve has a prevalence of 0,4% in the general population and 1,7% in the population over 65 years old (8). The pathophysiology of CAS is complex, it involves lipoprotein deposition, inflammation and osteoblast transition of valve interstitial cells (Hulin A et al. in this issue).

Risk factors for AS in the general population are the same as atherosclerotic vascular diseases, *i.e.*, diabetes, hypercholesterolemia, hypertension and tobacco usage (9).

Medication is unable to stop or revert the process of native aortic valve degeneration, with solutions limited to reparation/reconstruction or, in most cases, replacement.

### Aortic Valve Replacement

Worldwide the number of aortic valve replacement in 2003 was 290,000 and by 2050 is predicted to be 850,000 (10).

Prosthetic aortic valves can be of 3 different types: (1) Surgical Mechanical Aortic Valves in different material, including stainless steel, pyrolitic carbon or ceramic, and with different shapes - caged-ball, monoleaflet and bileaflet. They are structurally robust and can theoretically have a long service life (25–30 years). (2) Surgical Biological Aortic Valves are made of biological tissue that can be xenogenic (bovine or porcine) or allogenic (homograft), stented or stentless. Durability is the main problem with these valves, which last between 10–15 years. (3) Transcatheter or Percutaneous Aortic Valves are tissue heart valves and can be of two types: expanded over a balloon or self-expandable. They are inserted percutaneously and are easy to implant, but, like surgical bioprosthesis, they are not long lasting.

The surgical procedure for aortic valve replacement involves an open-heart surgery, the heart is stopped and the patient is attached to a bypass to oxygenate the blood. Since SAVI is quite invasive, it has been slowly replaced by TAVI, which can have 3 different sites of vascular access: transfemoral, subclavian or carotid artery and clinical trials are currently ongoing to evaluate the approach that will give least complications. TAVI is performed in cases where patients are at high risk of death during surgery, due to old age or the presence of additional diseases. Two randomized prospective clinical trials, PARTNER 1 (Placement of AoRtic TraNscatheER) (11) and CoreValve (12) have proven the superiority of TAVI over SAVI in a high-risk cohort of patients. Moreover, in July 2017 - the year of the 15 year anniversary of TAVI (13) - the FDA, based on the favorable conclusions of two trials, the PARTNER 2 (14) and the SURTAVI, has approved the use of TAVI in patients with intermediate risk of a negative outcome during open-heart surgery. But SAVI still remains the reference method, especially in low-risk patients. To be able to extend TAVI to all patients, regardless of surgical risk, more studies, focused on the outcomes of the procedure in the long run, are needed (15).

Both SAVI and TAVI are associated with thrombosis (2), but it is becoming evident that during TAVI there are more periprocedural ischemic and embolic strokes, caused by the dislodgement of debris from the aortic arch, annulus, and native valve (16). To reduce such thromboembolic events, the clinical trial GALILEO (clinicaltrials.gov, NCT02556203) is at the moment recruiting patients to test the hypothesis that, being thrombin a key-player in the pathophysiology of thromboembolic events, patients would benefit from treatment with anticoagulants, like rivaroxaban.

### Management of Aortic Valve Replacement

For an aortic valve replacement medical doctors are faced with many decisions, *e.g.*,: define when the aortic valve condition is severe enough to perform the replacement; what kind of intervention - SAVI or TAVI - to perform; and what kind of prosthetic aortic valve to use - mechanical or biological [(17)  ESC Guidelines]. This is why it is very important that a multidisciplinary heart team evaluates risks and benefits of all pre and post-procedural decision (18).

Usually, mechanical valves, which are more thrombogenic, but more durable, are implanted in patients younger than 65 years old, which have good hemodynamics, while biological valves are used mainly in the elderly. Although less thrombogenic, tissue valves (surgical or trasncatheter) are prone to structural valve deterioration (SVD), caused mainly by calcification (19) . Nevertheless, more than half of valve replacements are bioprosthetic, especially after the introduction of TAVI in 2007.

Since mechanical valves are thrombogenic, they require long-term vitamin K antagonists (warfarin) and antiplatelet drugs (aspirin) administration. However, such treatments may increase the risk of bleeding. Bioprosthetic, both surgical and transcatheter, valves have better hemodynamic properties compared to mechanical ones, therefore, the antithrombotic treatment is just required for the first months post-surgery (3–6 months) to reduce thromboembolic complications, during the process of prosthesis endothelialization (neointimal coverage of the frame and leaflets) (2).

The choice of the best valve to implant depends mainly on two risk factors: anticoagulation-related bleeding and valve deterioration. Tissue valves are implanted when the risk of bleeding with anticoagulation treatment is high, while mechanical valves are implanted when valve tissue deterioration could be accelerated, *i.e.*, in younger patients.

Antithrombotic management slows down, but does not eliminate the risk of prosthetic valve failure, which depends also on the life-style of the patient as well as on the pre-procedural metabolic profile and inflammatory status, especially for TAVI.

### Causes of Failure of Prosthetic Valves

Prosthetic valve dysfunction depends on the valve that has been implanted and on the procedure (SAVI or TAVI). Atherosclerosis risk factors, like diabetes, smoking, hypercholesterolemia, metabolic syndrome may accelerate failure of prosthesis, while dental procedures and other surgeries may increase the risk of valve infection.

#### Infection

The risk of infection of the prosthetic aortic valve is much higher compared to native valves and can affect all types of prosthesis equally, leading to infective endocarditis. Infections can arise just after surgery (within a week to one month post-surgery), or appear long after surgery (after 6 months). Periprocedural and 30 days infections are more common during SAVI compared to TAVI, although such differences are not statistically significant (7).

Since bacteria colonization of prosthetic valves and, of biomedical devices in general, is difficult to fight, because of biofilm formation (bacteria in the biofilm are more resistant to the usual antibiotics doses), it is important to prevent infections that can arise during dental procedures or other surgeries, with the use of antibiotics [(20) ESC Guidelines].

#### Thrombosis

Altered local flow due to the presence of prosthetic valve may be a trigger for thrombosis. In fact, high shear stress levels could potentially damage red blood cells (hemolysis) and activate platelets, promoting thrombogenesis.

The disruption of the normal local flow can also be caused by implantation errors *e.g.*, when the transplanted valve does not have the right geometry/model, with a specific annulus size, different for each patient. We talk in this case of a Patient-Prosthesis Mismatch (PPM). To prevent PPM cases, medical doctors should use fluid dynamic computational simulations before valve implantation.

While bioprosthesis are the least thrombogenic, mechanical and transcatheter valves are comparable in terms of thrombogenic potential, due to their similar transvalvular flow gradients (21).

Another important mechanism leading to thrombosis is surface–induced thrombosis, which has been well described in mechanical valves and other medical devices. The contact of prosthetic valves with blood (biomaterial-blood interaction) triggers a thrombogenic process that involves: (1) adhesion of platelets via surface-adsorbed plasma proteins, like lipoproteins, fibrinogen, fibronectin, von Willebrand factor (VWF) or laminin. (2) Activation of the “Contact Activation Coagulation System” via negatively charged surfaces activating Factor XII (FXII). (3) Activation of the “Extrinsic Coagulation System” via adhered microparticles containing Tissue Factor (TF), released by several activated cellular components, like activated leukocytes. (4) Adhesion of leukocytes, in particular neutrophils and neutrophil extracellular traps (NETs), leading to inflammatory reactions, which promote platelet capture and aggregation. (5) Activation of complement via FXII, which further amplifies the coagulation cascade. All these events result in thrombin generation, activation of platelets and formation of platelets-fibrin networks on the prosthetic surface. The fate of such thrombus would be to obstruct blood flow in the place that it was generated or to detach and enter the circulation. To counteract thrombus formation, macrophages can infiltrate the thrombus for the clearance of NETs and provide plasminogen activator, important for fibrinolytic processes (22).

With the more frequent use of transcatheter aortic valves it is becoming important to understand the pathological processes and the triggering mechanisms associated with thrombosis of such valves. In fact, thrombo-embolic events have been reported in TAVI patients especially in the first 3 months post-procedure. One hypothesis is that, since the native valve is not removed, but left in place during TAVI, the leaflets of the stenotic native valve are still rich in TF, which exacerbates platelet activation (2).

#### Calcification

Calcification occurs more on bioprosthetic valves than on mechanical valves. Bioprosthesis are made of glutaraldehyde-fixed porcine valve cusps or bovine pericardium, composed of devitalized cells valvular interstitial cells (VICs) or fibroblast from porcine or bovine tissues, respectively, embedded in an extracellular matrix of collagen, elastin, and glycosaminoglycans (GAGs). Glutaraldehyde is the primary cause of calcification (23). Although the pathophysiology of valve mineralization is poorly understood, collagen and elastin fibers can serve as nucleation sites for calcium phosphate minerals. Moreover, calcium phosphate minerals have also been observed at the membrane of devitalized VICs (24). The mechanism of formation of calcium deposits in devitalized cells is probably due to calcium influx from the surrounding area of the cells to the inside of cells. The consequence is the formation of hydroxyapatite by reaction of such Ca^2+^ with free phosphate groups derived from membrane’s phospholipids.

Procoagulant actors, such as phosphatidylserine-exposing activated platelets and TF-expressing immune cells or microparticles, lipid accumulation and inflammation may also play a role in calcification. However, the relationship between bioprosthesis calcification, lipids, inflammation, and thrombosis has never been established. Whether thrombosis promotes calcification, and/or *vice versa* is unknown.

### Outcomes: Challenges and Solutions

Considering all complications of prosthetic aortic valves, there is an urgent need to improve their design, biocompatibility and durability (25).

The development of a prosthetic aortic valve is a very complex matter, achieved with teams of chemists, bioengineers and medical doctors. A prosthetic aortic valve to be clinically safe and durable has to comply to many regulations, pass extensive *in vitro* testing, preclinical studies in animal models (pig or sheep) (Table 1) and clinical trials (26).

**Table 1:** *In vitro* and *in vivo* tests in prosthetic aortic valve development.

Biocompatibility	ISO 10993 tests
Infection	Anti-fouling tests (ISO 14160)
Hemodynamics	Pulse Duplicator (ISO 5840)
Durability	Durability Testers + Shelf life testing (ISO 5840–1 Annex G, H, I, J)
Calcification	*In vivo* animal models 20 weeks (ISO 5840–2)

Biocompatibility and haemocompatibility of the material of the valves is crucial and has to follow the ISO 10993 guidelines. The *in vitro* tests should evaluate the effect of the prolonged contact of the prosthetic valve surface with whole blood at 37°C under shear stress. Lysis of red blood cells can be measured using Lactate Dehydrogenase (LDH) activity, while flow-induced platelet activation can be studied in a perfusion chamber or in a cone and plate device.

An important parameter to determine is the clotting time of plasma that has been in contact with the biomaterial. Such test, if performed using specific inhibitors, allows the discrimination between intrinsic and extrinsic pathways of coagulation, which is important if we want improve prosthetic valve surfaces. Other important tests are cell toxicity as well as immunogenicity of the biomaterial of the valves. The latter evaluated is a measurement of complement (C5a and C3a) activation.

Anti-fouling properties refer to capacity of the material repulse bacteria or other microorganisms. With an anti-fouling biomaterial, microorganisms are not able to adhere and form biofilms of the surface of the implanted prosthesis. Biomaterials with anti-fouling properties would avoid colonization and accumulation of microorganisms on the surface of the valve (27). Examples of anti-fouling surfaces are poly(ethylene glycol) PEG, oligo(ethylene glycol) or zwitteronic species (28).

Geometry/Design of prosthetic aortic valves is of crucial importance to retain similar hemodynamic properties of native valves. Despite years of studies on the geometrical design of mechanical valves, the super-physiological shear stresses leading to valve deterioration, thrombosis and to a lesser extend calcification, are still detected with this kind of valves. Usually, hemodynamics of prosthetic valves are first tested *in silico*, using numerical simulations, like 2D computational fluid dynamic (CFD) or, more recently, 3D fluid-structure interaction (FSI) simulations. Hydrodynamic performance of a prosthetic valve is then evaluated *in vitro* using a Pulse Duplicator (ISO 5840:2005).

Durability is a critical issue, especially with bioprosthetic valves. An ideal bioprosthetic valve should be like a native valve, extremely durable, going through 40 million cycles a year and 3 billion during a life-time. In native valves durability and strength is given by the flexibility and heterogeneity of the supportive structures (collagen, connective tissue and elastin) and cells (Valvular Endothelial, VECs, and Interstitial cells, VICs). Bioprosthetic valves are far from having similar durability, making this issue an important point to improve for the next generation of bioprosthetic valves. Durability or prolonged accelerated wear testing is mandatory. Prosthesis durability testers can simulate 10 years of valve usage in 6 months.

Sterility is fundamental for implantable medical devices. Sterility is evaluated using the Sterility Assurance Level (SAL), which represents the probability of a single viable microorganism occurring on an item after sterilization. While this probability can be reduced to a very low number, it can never be reduced to zero. Accepted SAL values are 10^−3^ and 10^−6^ for non-implantable device and implantable device respectively. The methods used to sterilize are ethylene oxide, radiation (gamma rays), ozone or addition of antibiotics. It is important to choose the right sterilization method, as it can affect SVD.

## Conclusions and Future Perspective

Although extensive *in vitro* and *in vivo* testing is done prior to releasing a prosthetic valve on the market, prosthetic valve thrombosis, as well as infection and calcification, cannot be avoided.

Several solutions have been proposed to mitigate calcification, like chemical anticalcification agents like deritatives of ammino oleic acid (AOA). Such delipidating agent has been proven effective in removing membrane-bound phospholipids derived from devitalized cells and in reducing calcification (29).

Moreover, alternatives to glutaraldehyde fixation, which is the most used cross-linking agent, have also been proposed (dye-mediated photofication,carbodiimide-based fixation). In fact, glutaraldehyde residues in the bioprosthesis have been implicated in calcification and lack of endothelization (29).

Prevention of prosthesis failure could be achieved with the new generation of smart heart devices, capable of auto-detecting their status or by measuring specific markers in plasma that could predict prosthetic valve failure. For bioprosthesis, for example, several markers have been identified as predictors of SVD: the ratio apolipoprotein B and A-I (apoB/apoA-I) (30); Lipoprotein-associated phospholipase A2 (Lp-PLA) (31); and the ratio of oxidized low-density lipoprotein and high-density lipoprotein (OxLDL/HDL) and proprotein convertase subtilisin/kexin 9 (PCSK9) levels (32).

Lots of hopes lie in Heart Valve Tissue Engineering (HVTE), involving *in vitro* coating of a matrix with appropriate cell types. The matrix can be biodegradable or not and the cell types can be stem or progenitor cells, autologous or allogenic (10). The idea is to develop heart valve substitutes containing living cells able to actively respond and adapt to surrounding mechanical stresses, mimicking more closely the complex functions of native valves (33, 34).

Another alternative is Polymeric Heart Valves (PHV), primarily made of polyurethane (PU-PHV) (35). The geometry of such valves is better controlled (trileaflets) for optimal durability and hemodynamics. Since PU-PHVs are not made of animal tissue, they are safer and less expensive and could be used in TAVI, due to their flexibility. On the other hand, the creation of a flexible polymeric material that can withstand aortic valve flows has proven challenging and resulted in many failures.

To solve geometry issues, like PPM, the latest technologies use stereolithographic 3D printing of models based on X-ray computer tomography (CT) scans of native valves (36). Using this technology it becomes possible to produce a tailor-made prosthetic valve, made of tissue or polymers that would mimic closely the native valve with a minimal impact.

## Author Contributions

All authors listed have made a direct contribution to the manuscript and have approved it for its publication. LM wrote the manuscript. PL, NJ, AH and AN provided intellectual contributions and edited the manuscript. CO drafted and revised the manuscript.

## Conflict of Interest Statement

The authors declare that the research was conducted in the absence of any commercial or financial relationships that could be construed as a potential conflict of interest.
